# Spectroscopic and computational investigation of actinium coordination chemistry

**DOI:** 10.1038/ncomms12312

**Published:** 2016-08-17

**Authors:** Maryline G. Ferrier, Enrique R. Batista, John M. Berg, Eva R. Birnbaum, Justin N. Cross, Jonathan W. Engle, Henry S. La Pierre, Stosh A. Kozimor, Juan S. Lezama Pacheco, Benjamin W. Stein, S. Chantal E. Stieber, Justin J. Wilson

**Affiliations:** 1 Los Alamos National Laboratory, Los Alamos, New Mexico 87545, USA; 2 Stanford University, Stanford, California 94305, USA; 3 California State Polytechnic University, Pomona, California 91768, USA; 4 Cornell University, Ithaca, New York 14853, USA

## Abstract

Actinium-225 is a promising isotope for targeted-α therapy. Unfortunately, progress in developing chelators for medicinal applications has been hindered by a limited understanding of actinium chemistry. This knowledge gap is primarily associated with handling actinium, as it is highly radioactive and in short supply. Hence, Ac^III^ reactivity is often inferred from the lanthanides and minor actinides (that is, Am, Cm), with limited success. Here we overcome these challenges and characterize actinium in HCl solutions using X-ray absorption spectroscopy and molecular dynamics density functional theory. The Ac–Cl and Ac–O_H_2_O_ distances are measured to be 2.95(3) and 2.59(3) Å, respectively. The X-ray absorption spectroscopy comparisons between Ac^III^ and Am^III^ in HCl solutions indicate Ac^III^ coordinates more inner-sphere Cl^1–^ ligands (3.2±1.1) than Am^III^ (0.8±0.3). These results imply diverse reactivity for the +3 actinides and highlight the unexpected and unique Ac^III^ chemical behaviour.

## Abstract (web summary)

Actinium-225 is a promising isotope for α-therapy but progress in developing its chemistry is hindered by its high radioactivity and short supply. Here, the authors characterize actinium coordination in HCl solutions using X-ray absorption spectroscopy and molecular dynamics density functional theory.

## Graphical abstract

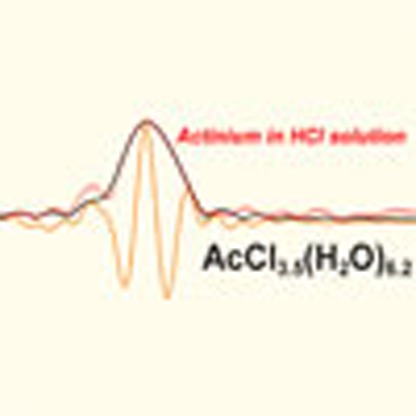

Understanding of the chemistry of trivalent actinides has broad-potential impacts on society through diverse areas, including cancer therapy^1^,^2^,^3^, nuclear waste^4^,^5^,^6^, energy and global security^7^. However, with the exception of U^III^ (refs 6, 7, 8, 9), fundamental understanding of the early-to-mid-actinides in the trivalent oxidation state (An=Ac^III^, Np^III^, Pu^III^, Am^III^, Cm^III^, Bk^III^ and Cf^III^) remains substantially less developed than that of the main group, transition metals and lanthanides. Significant experimental challenges associated with the highly radioactive samples, limited access to material of interest, and the redox instability of some An^III^ ions have necessitated numerous heroic efforts to advance An^III^ chemistry^6^,^10^,^11^,^12^,^13^,^14^,^15^.

The 2015 Long Range Plan for the DOE-NP Isotope Program^16^ highlighted the importance of advancing Ac^III^ chemistry in comparison with the other An^III^ ions. In this document, ^225^Ac was identified as a promising anticancer therapeutic agent. The report focused on global interest in exploiting the α-emissions from ^225^Ac and its radioactive daughters to selectively destroy the malignant cells^17^,^18^. To use these α-particles therapeutically, the ^225^Ac isotope must be attached to cancer-selective targeting vectors using a stable chelating agent. Unfortunately, the limited availability of ^225^Ac [t_½_=10.0(1) d; National Nuclear Data Center (NNDC)], or a useful surrogate, has impeded development of actinium chelators. For instance, the use of ^227^Ac, the longest-lived actinium isotope [t_½_=21.772(3) y; NNDC] provides the best possible alternative for improving ligand design. However, the worldwide inventory of ^227^Ac is currently insufficient for meeting this demand. Historically, these challenges were magnified by the electronic ground state of Ac^III^ (5*f*^0^ 6*d*^0^), which is essentially invisible to common spectroscopies (for example, ultraviolet–visible, fluorescence, electron paramagnetic resonance and so on). Hence, applying alternative characterization methods that account for (1) high radioactivity, (2) small samples sizes (1–30 μg) and (3) closed shell electronic configurations would facilitate innovative chelation designs for delivery of ^225^Ac through the biological milieu to the targeted tumour site.

Recent upgrades at the Stanford Synchrotron Radiation Lightsource (SSRL), such as the new 100-element solid-state Ge detector array on beamline 11-2, have provided new opportunities to overcome the aforementioned challenges. This capability has improved sensitivity for trace-level detection in environmental samples using X-ray absorption fine-structure (XAFS) spectroscopy^19^,^20^. While this instrumentation has been used primarily in environmental sciences, here we exploit the capability to conduct the first actinium XAFS study reported to date. Using this method, ^227^Ac in concentrated HCl solutions are characterized at the Ac L_3_-edge. Our interpretations of the spectra are guided by molecular dynamics density functional theory (MD-DFT) calculations^21^,^22^,^23^,^24^,^25^. To establish confidence in the spectroscopic and computational methods, the Ac^III^ results are compared with analogous measurements on Am^III^. This ion is selected because—among the redox stable trivalent actinides—its ionic radius is most similar to Ac^III^. Overall, this approach enables the Ac–Cl and Ac–O_H_2_O_ distances to be measured and allows the coordination chemistry of Ac^III^ and Am^III^ to be directly compared. The experimental and computational data show more chloride ions in the inner coordination sphere for Ac^III^ and the results are presented in comparison with previous actinide(III) XAFS studies. This study reveals an unexpected divergence in +3 *f*-element reactivity. Furthermore, the research highlights the need to better characterize An^III^ chemistry in support of developing α-emitting therapeutic agents.

## Results

### X-ray absorption near-edge structure spectroscopy

**Figure 1:**
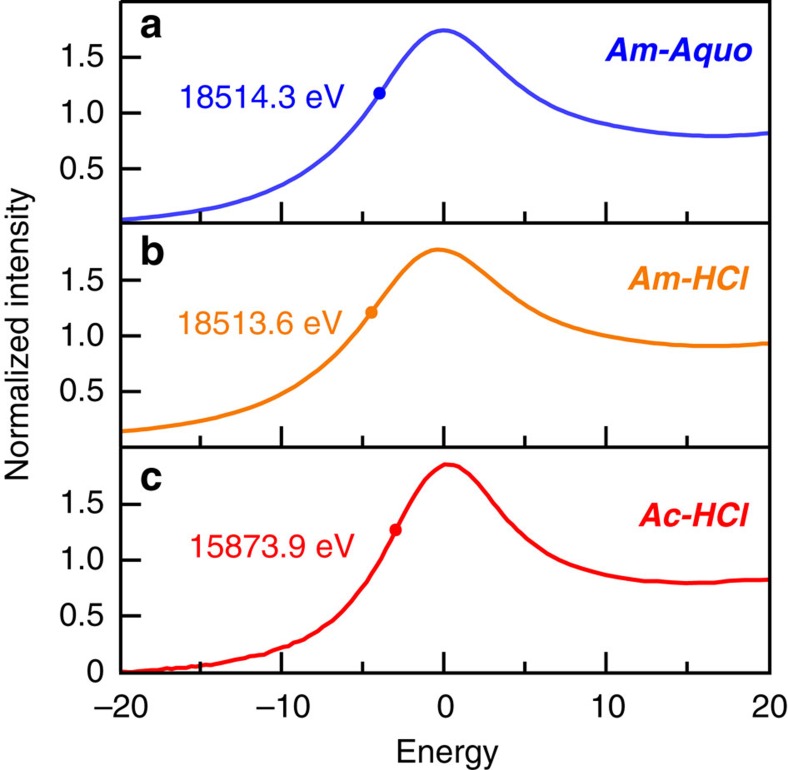
Solution phase Ac^III^ and Am^III^ L_3_-edge XANES. (**a**) XANES spectra for ***Am-Aquo*** (0.11 M HO_3_SCF_3_) in blue trace. (**b**) XANES spectra for ***Am-HCl*** (11 M HCl) in orange trace. (**c**) XANES spectra for ***Ac-HCl*** (11 M HCl) in red trace. The actinium and americium spectra were calibrated *in situ* to RbCl pellet (15,203.81 eV) and Zr foil (17,998 eV) respectively.

To evaluate Ac^III^ and Am^III^ binding of Cl^1–^ versus H_2_O, three solution-phase samples were prepared: actinium chloride (Ac^III^ in HCl; 11 M); americium chloride (Am^III^ in HCl; 11 M); and the americium aquo ion (Am^III^ in HO_3_SCF_3_, 0.11 M), referred to hereafter as ***Ac-HCl***, ***Am-HCl*** and ***Am-Aquo***. To mitigate safety concerns associated with the ^227^Ac isotope, radioactive daughters (primarily ^227^Th and ^223^Ra) were removed before the measurements at the SSRL synchrotron, as previously described (see Methods)^26^,^27^. The samples were then returned to Los Alamos National Laboratory before a significant quantity of the daughters re-emerged (∼10 days). The Ac and Am L_3_-edge X-ray absorption near-edge structure (XANES) spectra from ***Ac-HCl***, ***Am-HCl*** and ***Am-Aquo*** were background subtracted and normalized (Fig. 1). The spectra were similar as they contained a pronounced edge-peak superimposed on an absorption threshold. From the perspective of the free ion, the edge-feature could be crudely described as originating from electric-dipole allowed transitions from the actinide 2*p*-orbitals to unoccupied states that contain actinide 6*d*-character, that is, for Ac^III^ 2*p*^6^…5*f*^0^ 6*d*^0^→2p^5^…5*f*^0^ 6*d*^1^ (refs 28, 29).

The Ac L_3_-edge spectrum was of quintessential significance, as it represented the first actinium XANES measurement. The edge-peak line shape was quite broad and spanned over ∼30 eV, owing to the short core-hole lifetime. The peak maximum and inflection point (where first and second derivatives of the data equalled zero) were determined to be 15,877.3 and 15,873.9 eV, respectively. For the americium samples, the inflection point from ***Am-Aquo*** was found at 18,514.3 eV, which was consistent with americium in the +3 oxidation state^30^,^31^. Changing the solution matrix from HO_3_SCF_3_ (0.11 M) to HCl (11 M) shifted the edge-peak inflection point to lower energy by 0.7 eV. The Am L_3_-edge energy difference was attributed to an electronic change that accompanied Cl^1–^ displacement of H_2_O in the Am^III^ inner coordination sphere (*vide infra*), that is, ***Am-Aquo*** versus ***Am-HCl***. Given the relative ease with which the Ac^III^ and Am^III^ L_3_-edge XANES measurements were obtained—and in light of the incompatibility of other spectroscopic techniques for probing dilute actinium samples—we anticipate that these results will provide a foundation for characterizing the coordination chemistry of actinium in other chemical environments in the future.

### Extended X-ray absorption fine structure

**Figure 2:**
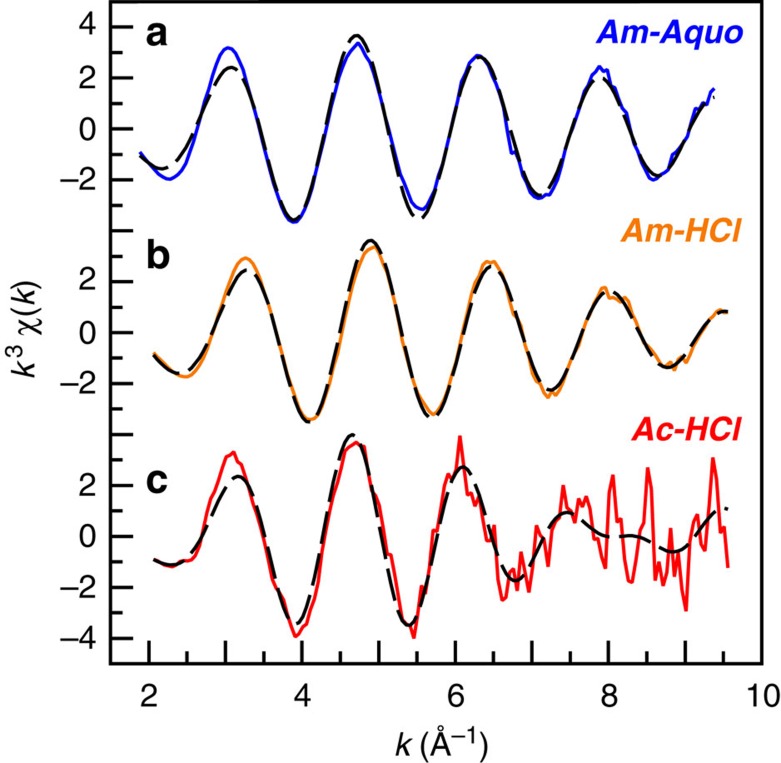
Solution phase Ac^III^ and Am^III^ L_3_-edge EXAFS. (**a**) EXAFS *k*^3^χ(*k*) spectra for ***Am-Aquo*** (0.11 M HO_3_SCF_3_) in blue trace. (**b**) EXAFS *k*^3^χ(*k*) spectra for ***Am-HCl*** (11 M HCl) in orange trace. (**b**) EXAFS *k*^3^χ(*k*) spectra for ***Ac-HCl*** (11 M HCl) in red trace. Fits to the data have been provided as dashed black traces.

Appreciable differences were observed in the solution-phase *k*^3^χ(*k*) extended X-ray absorption fine-structure (EXAFS) data (room temperature) from solutions containing ***Ac-HCl***, ***Am-HCl*** and ***Am-Aquo*** (Fig. 2). For instance, a substantial phase shift and change in frequency in the EXAFS oscillations was observed in the spectrum from ***Ac-HCl*** in comparison with the americium spectra. The ***Ac-HCl*** spectrum was comprised of at least two substantial contributions; one characterized by a long frequency and the other by a short frequency. The presence of two frequencies was most likely associated with a significant number of both Cl^1–^ and H_2_O molecules in the Ac^III^ inner coordination sphere. In contrast, for ***Am-HCl***, the frequency of EXAFS oscillations was dominated by one major sinusoidal component, such that the ***Am-HCl*** spectrum was almost superimposable on that from ***Am-Aquo***. The similarity between ***Am-HCl*** and ***Am-Aquo*** suggested that H_2_O molecules dominated the inner coordination sphere in ***Am-HCl***, with Cl^1–^ content being slight. Overall, this comparison highlighted significant differences in Ac^III^ versus Am^III^ solution speciation.

The EXAFS data were fit by allowing the coordination numbers (CN), the Debye–Waller factors (*σ*^2^), and the interatomic distances (*R*) to converge to reasonable values. Limitations associated with the solution-phase room-temperature EXAFS measurements constrained meaningful data to ∼10 *k* (9, 10.5 and 12 *k* for solutions containing ***Ac-HCl***, ***Am-HCl*** and ***Am-Aquo***, respectively). As a result, scattering pathways beyond the first coordination shell (including multiple scattering pathways, for example, Cl−M−H_2_O) were not considered. To most effectively describe how the EXAFS models were obtained, it was instructive to begin with ***Am-Aquo***, then introduce the possibility of Cl^1–^ ligation for ***Am-HCl***, and conclude with the ***Ac-HCl***.

**Figure 3:**
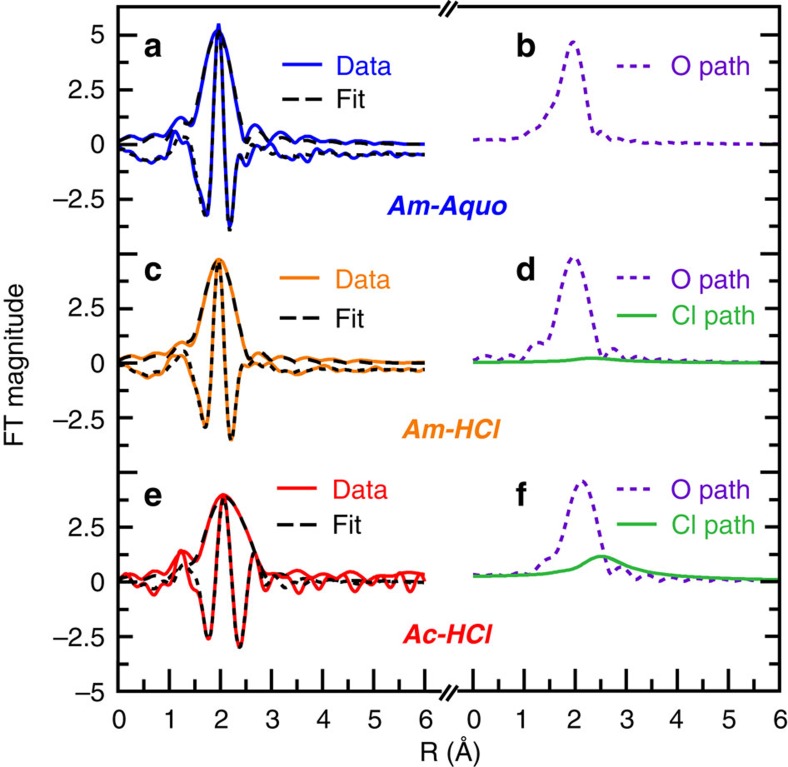
Fourier Transform of the Ac^III^ and Am^III^ *k*_3_-EXAFS. (**a**) Fourier Transform of *k*^3^-EXAFS spectra from ***Am-Aquo*** (0.11 M HO_3_SCF_3_) in blue trace and its fit in dashed black trace. (**b**) Oxygen path contribution in the fitted data in dashed purple. (**c**) Fourier Transform of *k*^3^-EXAFS spectra from ***Am-HCl*** (11 M HCl) in red trace and its fit in dashed black trace. (**d**) Oxygen and chlorine paths contribution in the fitted data in dashed purple and green solid trace, respectively. (**e**) Fourier Transform of *k*^3^-EXAFS spectra from ***Ac-HCl*** (11 M HCl) in red trace and its fit in dashed black trace. (**f**) Oxygen and chlorine paths contribution in the fitted data in dashed purple and green solid trace, respectively.

Before modelling the EXAFS data from ***Am-Aquo***, we initially calculated (FEFF8 (ref. 32)) an EXAFS spectrum using atomic coordinates obtained from the [Am(H_2_O)_9_][O_3_SCF_3_]_3_ single-crystal X-ray diffraction data, previously reported^33^. The calculated EXAFS spectrum was composed of two main scattering pathways. The first path originated from six oxygen atoms with an Am–O_H_2_O_ distance of 2.465 Å and the second path had three oxygen atoms at 2.578 Å. Although various EXAFS fitting parameters were considered based on these calculations, only one oxygen shell could be resolved in the experimental data, as our resolution between *k* of 2.6 and 12 was only 0.167 Å (*π*/2Δ*k*). The final model of the EXAFS data with the lowest residual factor and reduced chi-squared value consisted of 9.5±0.9 oxygen atoms at 2.48(1) Å (Table 1 and Figs 2 and 3). These Am–O_H_2_O_ bond distances and CNs were consistent with the Am(H_2_O)_9_^3+^ solid-state structure and EXAFS spectra previously obtained on Am^III^ aquo ions in different solvent matrices, for example, Stumpf *et al*.^34^ in 0.025 M HClO_4_ and Allen *et al*.^35^ in 0.25 M HCl (Table 1).

**Figure 4:**
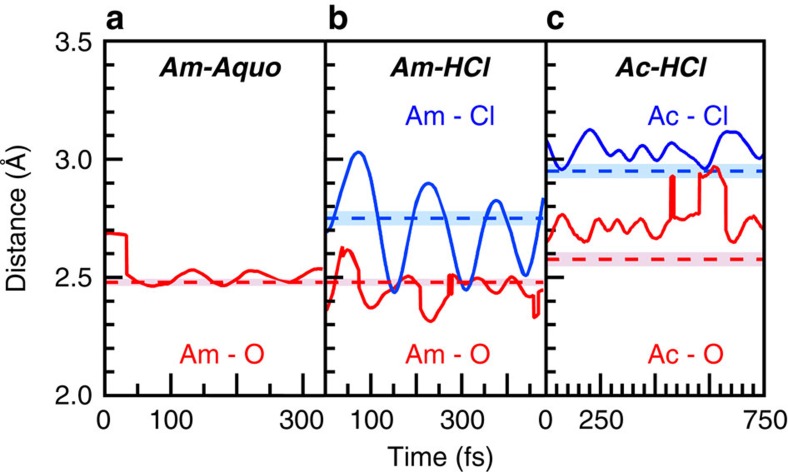
MDDFT calculations. The calculated mean M–O_H_2_O_ (solid red traces) and M–Cl (solid blue traces) distances for O and Cl atoms within 4 Å of the metal (Am or Ac) have been compared with the values obtained from the experimental EXAFS analyses (M–O_H_2_O_ dashed red and M–Cl dashed blue traces with their associated errors bars represented as shaded boxes). Experimental errors bars were 0.01 or 0.03 Å. See Table 1. (**a**) Data for ***Am-Aquo***, (**b**) Data for ***Am-HCl*** and (**c**) Data for ***Ac-HCl***.

The EXAFS fitting results can be interpreted using static or dynamic descriptions. A static interpretation suggested that the americium aquo ion had 9.5 H_2_O ligands held at a fixed 2.48(1) Å distance. In contrast, the dynamic interpretation described the EXAFS data as an averaged spectrum from a heterogeneous mixture over the lifetime of the experiment. In this scenario, the number of H_2_O molecules in the Am^III^ inner coordination sphere varied, as did the Am–O_H_2_O_ distances. To provide insight, MD-DFT calculations were carried out on a single Am^III^ ion inside a box (12.54 × 12.45 × 12.68 Å^3^) of explicit H_2_O (64 total) solvent molecules. Before the simulation, the components of the box were randomized (see Methods), and then the MD were modelled for 330 fs. The MD trajectory showed a time average of 8.1±0.3 H_2_O molecules within a 4 Å radius of the Am^III^ ion. The average Am–O_H_2_O_ distances were displayed by the red solid traces in Fig. 4. At the start of the calculations the variation in Am–O_H_2_O_ bond distances were large, ranging from 2.7 to 2.5 Å. However, after ∼50 fs, the magnitude of these oscillations became smaller and centred ∼2.5 Å. In general, these calculations were in excellent agreement with the EXAFS experiment. Both theory and experiment identified a CN around nine and the calculated average Am–O_H_2_O_ distances bracketed the experimental value (red dashed trace). The good agreement of the ***Am-Aquo*** experimental and computational EXAFS analyses with the single-crystal and solution-phase EXAFS studies provided confidence and credibility that our methods could be applied accurately to other actinide systems.

To obtain atomic coordinates for modelling the ***Am-HCl*** data, we calculated EXAFS spectra using FEFF8 from two different sources. The first model employed atomic coordinates from previously reported [AmCl_2_(H_2_O)_6_]Cl single-crystal X-ray diffraction data^36^, which had two Cl^1–^ and six H_2_O inner-sphere ligands. The second simulation utilized a DFT-optimized structure of AmCl_2_(H_2_O)_4_^1+^. Refinement of the experimental EXAFS data using fitting parameters from either model converged to the same point (Table 1 and Figs 2 and 3), showing an approximate nine-coordinate Am^III^ ion with 0.8±0.3 Cl^1–^ and 8.3±0.9 H_2_O molecules in the inner sphere. Although the contributions from Cl^1–^ to the overall EXAFS spectrum seemed slight (Fig. 3), omitting the Cl^1–^ shell provided a significantly worse model, by over 91% confidence as determined from the Hamilton statistical test^37^. The measured americium coordination environment seemed reasonable when compared with other solution-phase EXAFS studies. For instance, our ***Am-HCl*** CNs—collected in HCl solutions of 11 M—were bracketed by those determined from solutions with lower and higher Cl^1–^ concentrations (Table 1). Allen *et al*.'s EXAFS experiments at lower HCl solutions (0.25 M) showed no inner-sphere Cl^1–^ ligands, while those at higher Cl^1–^ concentrations—a mixture of HCl (0.25 M) and LiCl (12.5 M)—showed 1.8 Cl^1–^ ligands^35^. When experimental errors were considered, our data also agreed with measurements conducted at similar Cl^1–^ concentrations, for example, Allen *et al*. identified 1.2 Cl^1–^ ligands in solutions containing mixtures of HCl (0.25 M) and LiCl (10.0 M) (ref. 35). Our 2.75(3) Am–Cl and 2.48(1) Am–O_H_2_O_ distances also agreed with metrics from the solid-state single-crystal X-ray diffraction study, 2.799(2) and 2.462(9) Å, respectively^36^, and were consistent with Allen *et al*.'s solution-phase EXAFS results in LiCl (10.0 M, 12.5 M); which had Am–Cl distances between 2.80 and 2.81 Å and Am–O_H_2_O_ distances varying from 2.48 to 2.51 Å^35^.

These ***Am-HCl*** EXAFS results agreed with the MD-DFT calculations. The calculations were conducted similarly to those described above for ***Am-Aquo***, with the exception that one Cl^1–^ anion was included in the calculation. Over the course of 480 fs, we calculated an average of 1 Cl^1–^ and 8.05±0.9 H_2_O molecules within a radius of 4 Å from the Am^III^ ion. The average Am–Cl and Am–O_H_2_O_ distances were quite similar to the experimental EXAFS values as shown in Fig. 4.

Given the paucity of actinium structural studies^38^,^39^, the DFT and MD-DFT calculations proved useful in guiding the interpretation of the EXAFS spectrum from ***Ac-HCl***. Atomic coordinates for the EXAFS refinement were obtained from MD-DFT calculations on AcCl_3_(H_2_O)_5_. The resulting EXAFS analysis identified two unique scattering paths; one associated with the H_2_O ligands composed of 6.6±1.8 molecules at 2.59(3) Å and a second at 2.95(3) Å consisting of 3.2±1.1 Cl^1–^ ligands (Debye–Waller factor=0.0074 Å^2^; Figs 2 and 3). These bond distances were in excellent agreement with the sum of the Ac^III^ and Cl^1-^ ionic radii, 1.12 Å+1.81 Å=2.93 Å (ref. 40). If the chlorine path was ignored and only the oxygen path used, the quality of the fit decreased (>93% confidence based on the Hamilton's statistical test^37^), gave a high Debye–Waller factor value (0.018 Å^2^), and provided an unrealistic number of coordinated H_2_O molecules (14 total).

The ***Ac-HCl*** experimental data agreed well with MD-DFT calculations conducted on a box containing one Ac^III^, three Cl^1–^ ligands and 64 H_2_O molecules for 1 ps. The MD-DFT revealed all three Cl^1-^ and 6.14±0.34 H_2_O ligands within 4 Å of the Ac^III^ ion for the duration of the calculation. The average Ac–O_H_2_O_ and Ac–Cl distances were calculated to be 2.76±0.08 and 3.04±0.13 Å, respectively. Although the magnitudes of these values were larger than that determined experimentally (by 0.17 Å for Ac–O_H_2_O_ and 0.09 Å for Ac–Cl), the calculated and experimental results agreed within 1*σ* for the Ac–Cl, and 2*σ* for the Ac–O_H_2_O_. Such deviations between theory and experiment were not unusual and have been observed in calculations on other systems that employed the generalized gradient approximation functional. The calculated CNs also agreed with the experimental values, showing ∼3 Cl^1–^ and ∼6 H_2_O inner-sphere ligands. We note that the agreement between experiment and theory was better in the two americium cases described above. It was possible that these subtle differences between theory and experiment resulted from the low signal-to-noise at high *k* in the experimental Ac L_3_-edge EXAFS spectrum, as the actinium concentrations were appreciably smaller than americium (μM versus mM). Alternatively, it was possible that the behaviour of Ac^III^ was more difficult to model, as the fundamentals of actinium have been less well defined than those of americium. Regardless, both theory and experiment indicated substantial differences between the Ac^III^ and Am^III^ coordination environments in HCl solutions, which represented an unexpected variation in actinide(III) reactivity. It is this observation that has inspired our future efforts to better understand the origin for diverging Ac^III^ versus Am^III^-binding preferences.

## Discussion

The experimental and computational results described here represented an exciting leap forward from previous actinium coordination chemistry studies. To most effectively communicate to the reader the significance of these results, we found it instructive to summarize the previous Ac^III^ coordination chemistry. The majority of actinium chemistry has been inferred from methodical radio-analytical and extraction studies conducted on trace-level quantities of actinium^39^,^41^,^42^,^43^. Although these previous efforts revealed differences between Ac^III^ and the other trivalent actinide and lanthanide elements, in general, they have been interpreted as suggesting that Ac^III^ is a hard oxophilic tri-cation, in direct analogy to other actinide(III) cations. In addition, there have been a few studies that used macroscopic quantities of Ac^III^ for analysis by ultraviolet–visible spectroscopy^43^,^44^. However, these spectra were essentially featureless—owing to the actinium 5*f*^0^ 6*d*^0^ ground-state electronic configuration—and did not provide an insight into the actinium coordination environment. The majority of actinium structural data was obtained during the Manhattan project and through the years shortly after World War II. For example, Fried *et al*.^38^ and Zachariasen^45^,^46^,^47^ deduced structures and cell parameters using X-ray diffraction patterns from crystalline powders of nine actinium compounds assumed to be AcF_3_, AcCl_3_, AcBr_3_, Ac_2_O_3_, Ac_2_S_3_, AcOF, AcOCl, AcOBr and AcPO_4_·H_2_O. Subsequently, Farr *et al*. used X-ray diffraction to characterize microgram quantities of a mixture of actinium metal and actinium hydride^48^. Years later, an attempt to prepare some inorganic compounds with 10 mg of ^227^Ac occurred. Unfortunately, radiation damage to samples and the diffraction films limited this effort to generate a single powder pattern of actinium oxalate, Ac_2_(C_2_O_4_)_3_·10H_2_O (ref. 49).

Reported here, we overcame some of the challenges associated with spectroscopic analyses of bulk actinium samples using new XAFS capabilities, which have been typically employed in trace-level environmental science studies. Briefly, we characterized the chemistry of Ac^III^ and Am^III^ in concentrated HCl (11 M) solutions and compared the results with solution-phase measurements from the americium aquo ion in dilute HO_3_SCF_3_ (0.11 M). Calculations involving MD-DFT, previously reported single-crystal X-ray diffraction, and previous solution-phase EXAFS results (when possible) were employed to guide our spectral interpretations. These efforts enabled the Ac–Cl and Ac–O_H_2_O_ distances to be directly measured for the first time. Moreover, the studies revealed a substantial difference between Ac^III^ and Am^III^ solution speciation. In concentrated HCl solutions, we observed that Ac^III^ and Am^III^ were coordinated by approximately nine ligands; however, Ac^III^ preferred approximately six H_2_O molecules and three Cl^1–^ ions, while Am^III^ was coordinated by approximately eight H_2_O and one Cl^1–^ ligands. It is well known that evaluating CN using EXAFS typically provides values with high uncertainty, ∼20%. In spite of the large errors associated with the Ac^III^ (CN_Cl_=3.2±1.1) and Am^III^ (CN_Cl_=0.8±0.3) chloride CNs, these values clearly indicated that Ac^III^ had more Cl^1-^ ligands in the inner coordination sphere than Am^III^. These results were indeed unexpected, for example, stability constants for Ac–Cl and Am–Cl were reported to be nearly identical^6^.

**Figure 5:**
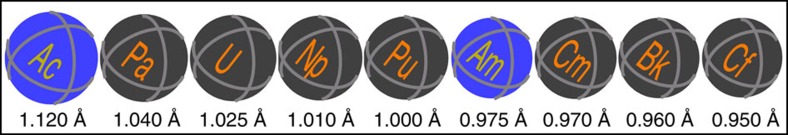
Ionic radii of the early to mid-actinides. Scheme showing the ionic radii (Å) of trivalent actinides from actinium to californium drawn to scale for six-coordinate ions^40^.

Perhaps differences in steric crowding accounted for the varied H_2_O and Cl^1–^ CNs for the large Ac^III^ versus smaller Am^III^ ions (Fig. 5). However, Ac^III^ electronic structure contributions may also contribute to difference in H_2_O versus Cl^1–^ binding. While the generality and origin of these results have yet to be established, they tempt us to revisit the traditional descriptions of Ac^III^. For example, consider the possibility that Ac^III^ is substantially less polarizing than the rest of the *f*-elements, which are well established as hard and oxophilic ions. In this sense, one might crudely compare the changes in chemical hardness from Ac^III^ to Am^III^ to that of iodine to fluorine. It is conceivable that a reclassification could provide insight into previous reports where actinium behaved substantially different than trivalent actinides and lanthanides^41^,^42^. Given that many Ac^III^ chelators are based on the coordination chemistry of lanthanides and minor actinides, better characterization of the long Ac–ligand bond distances, binding preferences, and speciation has the potential to improve design parameters for actinium chelators. In this context, the results here serve as motivation for future work focused on using XAFS spectroscopy and MD-DFT calculations to advance understanding of actinium reactivity with ligands that are more relevant to α-therapy development.

## Methods

### General consideration

Caution! The ^243^Am and ^227^Ac isotopes α-, β- and γ-emitting radionuclides have high specific activity and decay to α-, β- and γ-emitting isotopes. Hence, this research was conducted in a radiological facility with appropriate analyses of these hazards and implementation of controls for the safe handling and manipulation of these toxic and radioactive materials. All direct handling of these radionuclides was conducted within certified fume hoods and monitored with appropriate α-, β- and γ-particle detecting instruments. The ^227^Ac and ^243^Am isotopes were supplied by the United States Department of Energy Office of Science Isotope Program in the Office of Nuclear Physics. Hydrochloric acid and trifluoromethanesulfonic acid were obtained commercially (Fisher Scientific). Water was purified to 18.2 MΩ/cm resistivity using Thermo-Scientific Barnstead Nanopure or Millipore Nanopure water purification systems. Resins used for separations—DOWEX AG1-X8 (BioRad; 100–200; Cl form) and branched DGA (Eichrom)– were suspended in water, and the fines decanted before use. Separations were characterized using γ-spectroscopy using an EG&G Ortec Model GMX-35200-S HPGe detector system in combination with a Canberra Model 35-Plus multichannel analyser associated with Gamma Vision software. Characterization was also performed using α-spectroscopy with and Ortec Octete+ α-spectrometer associated with Ortec Alpha Vision analytical software to control the spectrometer, to acquire and analyse the data.

### Sample preparation

The coordination chemistry studies described herein made use of ^227^Ac (2 mCi; 28 μg) and ^243^Am (0.5 mg per sample). Safety concerns regarding the radiation dose from the ^227^Ac sample were mitigated by removing the ^227^Ac daughters (primarily ^227^Th and ^223^Ra) the day before shipping to the SSRL synchrotron facility and by completing the measurements before significant quantities of the ^227^Ac daughters re-emerged, ∼10 days from separation.

Given the scarcity of ^227^Ac and ^243^Am, these actinide isotopes were recycled after the XAFS data collections. In the case of actinium, the sample matrix was converted from HCl to HNO_3_ (8 M) and the actinium was recovered and purified using the chromatographic procedures described below^26^,^27^. For americium, samples were recovered and purified using cation exchange chromatography^50^. In general, the samples were dissolved in dilute acid (five drops HCl in 5 mL H_2_O) and loaded onto a Biorad column (10 ml) charged with AG50x8 resin (2 ml) that had been conditioned with HCl (1 M; 5 × 10 ml). The column was washed with HCl (0.1 M; 6 × 10 ml), and americium was eluted from the column using concentrated HCl (12 M; 5 × 5 ml). The recovered actinium and americium samples were analysed by γ-spectroscopy to confirm isotopic purities and the americium samples by ICP-AES to confirm chemical purities.

### Actinium-227 samples

**Figure 6:**
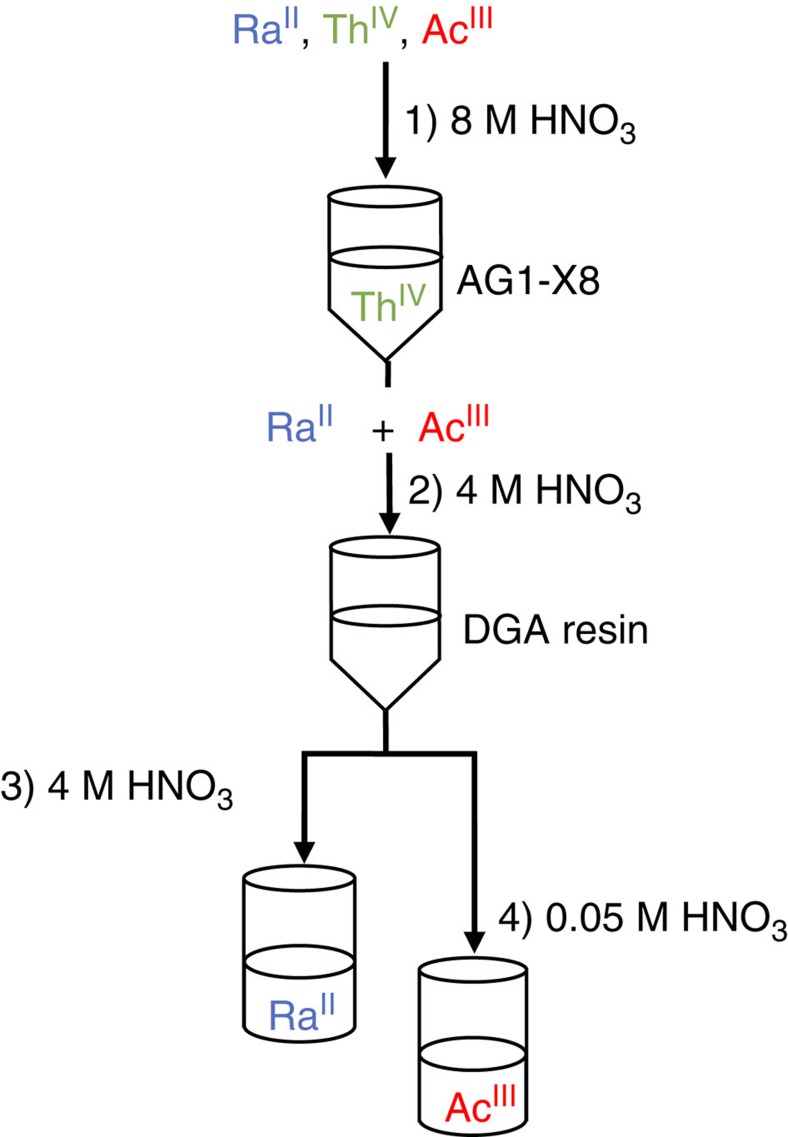
Cartoon describing ^227^Ac radiochemical purification. Scheme showing the procedure followed to separate ^227^Ac from its daughters ^229^Th and ^223^Ra using anion exchange resin and DGA resin, respectively.

Using previously established methods^26^,^27^, radiochemical purification of ^227^Ac was accomplished by exploiting oxidation state differences between the Ac^III^ parent and the tetravalent (Th^IV^) and divalent (Ra^II^) daughters (Fig. 6). The ^227^Ac isotope, and its daughters, were dissolved in HNO_3_ (1 ml; 8 M). The solution was loaded using HNO_3_ (4 × 1 ml; 8 M) onto a Biorad column charged with an anionic exchange resin (1 ml, AG1-X8, BioRad). The resin had been conditioned with H_2_O (5 × 3 ml), HNO_3_ (5 × 3 ml 8 M), H_2_O (1 × 2 ml) and HNO_3_ (3 × 3 ml 8 M). Under these conditions, ^227^Ac, ^223^Ra, ^211^Pb and ^211^Bi, eluted from the column during the load and with subsequent washes with HNO_3_ (10 ml; 8 M). Meanwhile ^227^Th was retained on the resin. The eluted ^227^Ac fractions were combined and diluted with water by a factor of two, such that the resulting solution contained HNO_3_ (4 M). The solution was loaded onto a Biorad column charged with branched DGA resin (1 ml). The resin has been conditioned with HNO_3_ (3 × 2 ml; 0.05 M) followed by HNO_3_ (3 ml; 4 M). Under these conditions, ^223^Ra eluted while ^227^Ac remained bound to the resin. The resin was washed with HNO_3_ (3 × 2 ml; 4 M). Subsequently, ^227^Ac was eluted using with HNO_3_ (4 × 2 ml; 0.05 M). The ^227^Ac concentration was determined by γ-spectroscopy to be highest in the first two fractions. The ^227^Ac fractions were combined and the solution evaporated in a conic shape glass vial on a hot plate under a slow stream of air to a soft dryness. The solid residue was converted from NO_3_^1−^ to Cl^1−^ to generate a solution of actinium in concentrated HCl by repeated evaporation and dissolution in HCl (11 M; 2 × 2 ml). The solid residue was then dissolved in HCl (0.455 ml; 11 M) and transferred to the XAFS holder.

### Americium-243 samples

Americium samples in either HCl (11 M) or HO_3_SCF_3_ (0.11 M) acidic solutions were prepared from a stock of AmO_2_ dissolved in HCl (6 M). Solutions of americium in HCl (11 M) were generated in direct analogy to the method described above for actinium. Briefly, an aliquot from an ^243^Am stock solution containing Am^III^ (0.6875, mg) in HCl (0.250 ml; 6 M) was heated to dryness in a glass vial in a sand bath on a hot plate. The solid was then dissolved in concentrated HCl (0.5 ml; 11 M) and this evaporation/dissolution process was repeated two more times. The resulting solid residue was dissolved (peach colour) in HCl (0.455 ml; 11 M) and transferred to the XAFS holder.

The solution containing americium aquo ion was prepared from an aliquot of an ^243^Am stock solution containing Am^III^ (0.6875, mg) dissolved in HCl (0.250 ml; 6 M). The solvent was removed by heating the solution in a glass vial in a sand bath on a hot plate. The solid residue was then dissolved in Millipore H_2_O and transferred to an Eppendorf tube (2 ml volume). Quickly, NaOH (0.050 ml; 2 M) was added, the solution shaken, and centrifuged for 3 min (6,000 r.p.m.). The supernate was decanted from the resulting pink precipitate and discarded. The solid was washed with NaOH (2 × 0.06 ml; 10 mM) and with Millipore H_2_O. The solid precipitate was then dissolved in trifluoromethanesulfonic acid (0.455 ml; 0.11 M) and transferred to the XAFS holder.

### XAFS Sample preparation

All XAFS samples were loaded into XAFS cells that were triply contained, which protected against release of radiological material during samples shipment and XAFS experiments. The XAFS holder consisted of a plastic holder body with a 2 mm well equipped with a set of Teflon and a Kapton windows (1 mil). Solutions were introduced into the holder through an injection hole sealed with a gasket that was held in place by an aluminium plate. The sample cell holder was then transferred into the secondary and the tertiary container, which were best described as a set of nested aluminium holders equipped with Kapton windows (2 mil).

### XAFS measurements

The XANES and EXAFS were measured at SSRL under dedicated operating conditions (3.0 GeV, 5%, 500 mA) on end station 11-2. This beamline was equipped with a 26-pole, 2 Tesla wiggler, utilized a liquid nitrogen-cooled double-crystal Si[220] monochromator and employed a collimating and focusing mirrors. A single energy was selected from the white beam with a liquid-N_2_-cooled double-crystal monochromator utilizing Si[220] (*ϕ*=0) crystals. The crystals were run de-tuned by 35%, ∼500 eV above the absorption edge to eliminate higher harmonics from the monochromatic light. The samples were attached to the beamline 11-2 XAFS rail. The rail was equipped with three ionization chambers through which nitrogen gas was continually flowed. One chamber was positioned before the sample holder, to monitor the incident radiation (*I*_0_, 10 cm). The second chamber was positioned after the sample holder, such that sample transmission (*I*_1_, 30 cm) could be evaluated against *I*_0_, while a third chamber (*I*_2_, 30 cm) positioned downstream from *I*_1_, so that the XANES of a calibration foil could be measured *in situ* during the XAFS experiments against I_1_. Conditions for the americium aqua ion sample was also optimized for transmission data collection. Actinium and americium solution samples in concentrated HCl were measured in fluorescence mode using solid-state 100-Ge element detector against the incident radiation (*I*_0_). Low-energy contributions to the fluorescence signal were removed using a bromine filter (three path lengths) for actinium and yttrium (three path lengths) for americium. Before measurements, dead-time correction measurements were performed at ∼400 eV above the element edge of the filter. The dead time correction curve corresponds to the plot of the windowed counts of the emission line of interest versus the total of incoming counts in the solid-state detector. This procedure was performed by putting filters (Se for Ac and Sr for Am) in front of the fluorescence detector.

### XAFS data analysis

Data manipulation and analysis was conducted as previously described^51^. First the data were dead-time corrected and calibrated to the energy of the first inflection point of the calibration foil measured *in situ*. The measurements were calibrated as follows. For actinium, a RbCl pellet was prepared by sandwiching RbCl diluted with BN to a one absorption length thickness. The energy for the first inflection point was determined in comparison with the Bi L_II_-edge (15,711 eV) to be 15,203.81 eV. Americium samples were calibrated to a zirconium foil at 17,998 eV. Data from the analytes were analysed by fitting a line to the pre-edge region, which remove the background from experimental data in the spectra. Then a second- to third-order polynomial fitting was chosen for the post-edge region. The difference between pre- and post-edge lines is set to unity at the first inflection point, normalizing the absorption jump to 1.0. Each of the samples were measured for several hours resulting in the collection of 25 scans for Ac and five scans for Am. Fittings using ATHENA and ARTEMIS^52^ were performed using crystallographic data from americium triflate^33^, americium trichloride hexahydrate^36^ and FEFF8 calculations^32^. The spectra were fitted using only single-scattering paths obtained from FEFF8. The adjustments of spectra were performed in different *k* and *R* ranges for each samples, where *R* is the distance to the neighbouring atom; americium triflate was adjusted in 2.6<*k*<12 Å^–1^ and 1.25<*R*<3 Å, americium in concentrated HCl in 2.6<*k*<10.5 Å^–1^ and 1.1<*R*<3.5 Å and actinium in concentrated HCl in 2.6<*k*<9 Å^–1^ and 1.2<*R*<3.45 Å. For the fitting procedure, the CN, the distance (*R*) and the Debye–Waller factor (*σ*^2^) were allowed to vary but for the last factor a single value was chosen for both paths used for the first shell. A single value of energy shift (Δ*E*_0_) was used for all scattering paths. The amplitude reduction factor (S_0_^2^) was set at 0.9 based on initial fits.

### MD-DFT calculations

The Born–Oppenheimer MD simulations in the Helmholtz ensemble (NVT) were performed using the computer code VASP (Vienna Ab-initio Simulations Package)^53^ version 5.35. In this code, the forces on the ions are calculated from the electronic structure of the whole system computed using DFT at the generalized gradient approximation level using the functional by Purdue–Burke–Enzerhof^54^. A simulation box of (12.54 × 12.45 × 12.68 Å^3^) was used, including the metal ion (M^3+^), three Cl^1−^ counter-ions maintained the simulation box neutral, and for solvent medium we used 64 water molecules. However, for the simulations of the single aquo ion no counter-ions were added to the system and a uniform background charge of −3 was added to keep the neutrality of the simulation box. The basis set consists in an expansion into plane-wave functions. Because of the large size of the simulation box, the *k*-space representation included only the Γ point. The energy cutoff for the plane-wave expansion was set at 500 eV and scalar relativistic effects were included using the PAW-PBE potentials^55^. Initially the metal ion and the closest neighbouring molecules and counter ions were kept frozen and the solvent plus remaining counter-ion atoms were heated up to 498 K to be thermalized for 1 ps. After that a 1 ps run was done at 298 K with all the degrees of freedom released to thermalize the complex with the solvent. Finally a 1 ps data collection run was performed where we monitored the solvent and ion dynamics.

### Data availability

The data that support the findings of this study are available within the article or from the corresponding authors on request.

## Additional information

**How to cite this article:** Ferrier, M. *et al*. Spectroscopic and computational investigation of actinium coordination chemistry. *Nat. Commun.* 7:12312 doi: 10.1038/ncomms12312 (2016).

## Figures and Tables

**Table 1:** Structural parameters determined using solution-phase EXAFS.

**Compound**	**Matrix**	**Scattering path**	**CN**	***R*** **(Å)**	***σ***^**2**^ **(Å**^**2**^)	**Δ*****E***_**0**_ **(eV)**
***Ac-HCl***	**HCl (11 M)**	**Ac–O**_**H_2_O**_**Ac–Cl**	**6.6±1.83.2±1.1**	**2.59(3)2.95(3)**	**0.00740.0074**	**−4.29**
U^III^(H_2_O)_*y*_ (ref. 56)	pH 0 HCl	U–O_H_2_O_	9.1±0.6	2.52(1)	0.009(1)	12.8
Np^III^(H_2_O)_*y*_ (ref. 56)	pH 0 HCl	Np–O_H_2_O_	10.0±1.2	2.51(1)	0.009(1)	7.2
Pu^III^(H_2_O)_*y*_ (ref. 35)	LiCl (0.01 M)	Pu–O_H_2_O_	9.2±0.33	2.51(2)	0.010	**−**10.4
Pu^III^(H_2_O)_*y*_ (ref. 56)	pH 0 HCl	Pu–O_H_2_O_	9.9±0.3	2.49(1)	0.009(1)	7.0
Pu^III^_aq_ (ref. 57)	HClO_4_ (1 M)	Pu–O_H_2_O_	8.6±0.2	2.50(2)	0.0083	7.16
***Am-Aquo***	**HO**_**3**_**SCF**_**3**_ **(0.11 M)**	**Am–O**_**H_2_O**_	**9.5**±**0.87**	**2.48(1)**	**0.0088**	**−4.71**
Am^III^(H_2_O)_x_^3+^ (ref. 34)	HClO_4_ (0.025 M)	Am–O_H_2_O_	8.3±0.4	2.473(4)	0.0071(6)	**−**12.2
Am^III^(H_2_O)_x_^3+^ (ref. 35)	HCl (0.25 M)	Am–O_H_2_O_	10.3±0.33	2.48(2)	0.009	**−**8.7
Am^III^(H_2_O)_*x*_^3+^ (ref. 58)	NaClO_4_ (0.03 M, pH 3.5)	Am–O_H_2_O_	9.0±0.0	2.47(1)	0.0074(5)	7.2
***Am-HCl***	**HCl (11 M)**	**Am–O**_**H_2_O**_**Am–Cl**	**8.3**±**0.860.8**±**0.3**	**2.48(1)2.75(3)**	**0.00950.0095**	**−4.29**
Am^III^Cl_*x*_(H_2_O)_*x*_ (ref. 35)	HCl (10 M LiCl)	Am–O_H_2_O_Am–Cl	7.6±0.331.2±0.1	2.48(2)2.80(6)	0.0090.005	**−**7.3
Am^III^Cl_*x*_(H_2_O)_*x*_ (ref. 35)	HCl (12.5 M LiCl)	Am–O_H_2_O_Am–Cl	6.4±0.331.8±0.1	2.51(2)2.81(6)	0.0090.005	**−**6.1
Cm^III^(H_2_O)_*y*_ (ref. 35)	HCl (0.25 M)	Cm–O_H_2_O_	10.2±0.33	2.45(2)	0.009	**−**13.0
Cm^III^(H_2_O)_*y*_ (ref. 12)	HClO_4_ (1 M)	Cm–O_H_2_O_	7.0±0.4	2.469(7)	0.0071(8)	
Cm^III^Cl_*x*_(H_2_O)_*y*_ (ref. 35)	LiCl (12.3 M)	Cm–O_H_2_O_Cm–Cl	6.1±0.332.4±±0.1	2.45(2)2.76(6)	0.0090.005	**−**13.5
Bk^III^(H_2_O)_*y*_ (ref. 11)	HClO_4_ (1 M)	Bk–O_H_2_O_	9.0±0.6	2.43(2)	0.009(2)	2.7
Cf^III^(H_2_O)_*y*_ (ref. 59)	HClO_4_ (0.1 M)	Cf–O_H_2_O_	8.0±0.0	2.42(1)	0.0077(1)	1.76
Cf^III^(H_2_O)_*y*_ (ref. 60)	HCl (1 M)	Cf–O_H_2_O_	8.5±1.5	2.42(2)	0.0095(1)	1.4

CN, coordination number; EXAFS, extended X-ray absorption fine structure.

Comparison between data obtained in this study from ***Am-Aquo***, ***Am-HCl*** and ***Ac-HCl*** with previously reported An^III^ aquo and chloride complexes.

Data shown in bold are from this study.

## References

[b1] McDevitt M. R. . Radioimmunotherapy with alpha-emitting nuclides. Eur. J. Nucl. Med. 25, 1341–1351 (1998). 9724387 10.1007/s002590050306

[b2] Steinbach O. C. The latest developments in therapeutic delivery. Ther. Deliv 5, 113–118 (2014). 10.4155/tde.14.26 24998270

[b3] Parker S. . Alpha emitter radium-223 and survival in metastatic prostate cancer. N. Engl. J. Med. 369, 213–223 (2013). 23863050 10.1056/NEJMoa1213755

[b4] Mathur J. N., Murali M. S. & Nash K. L. Actinide partitioning—a review. Solvent Extr. Ion Exc. 19, 357–390 (2001).

[b5] Poinssot C., Rostaing C., Baron P., Warin D. & Boullis B. Main results of the French program on partitioning of minor actinides, a significant improvement towards nuclear waste reduction. Proc. Chem.y 7, 358–366 (2012).

[b6] Morss L. R., Edelstein N. M. & Fuger J. The Chemistry of the Actinide and Transactinide Elements Springer (2006).

[b7] Paviet-Hartmann P., Cerefice G., Stacey M. R. & Bakhtiar S. in *Proceedings of ICONE19, 19th International Conference on Nuclear Engineering* (Chiba, Japan, 2011).

[b8] Evans W. J. & Kozimor S. A. Expanding the chemistry of U^3+^ reducing agents. Coord. Chem. Rev. 250, 911–935 (2006).

[b9] Drozdzynski J. Tervalent uranium compounds. Coord. Chem. Rev. 249, 2351–2373 (2005).

[b10] Knope K. E. & Soderholm L. Solution and solid-state structural chemistry of actinide hydrates and their hydrolysis and condensation products. Chem. Rev. 113, 944–994 (2013). 23101477 10.1021/cr300212f

[b11] Antonio M. R., Williams C. W. & Soderholm L. Berkelium redox speciation. Radiochim. Acta 90, 851–856 (2002).

[b12] Skanthakumar S., Antonio M. R., Wilson R. E. & Soderholm L. The curium aqua ion. Inorg. Chem. 46, 3485–3491 (2007). 17407283 10.1021/ic061798b

[b13] Polinski M. J., Alekseev E. V., Depmeier W. & Albrecht-Schmitt T. E. Recent advances in trivalent *f*-element borate chemistry. Z. Kristallogr. 228, 489–498 (2013).

[b14] Cary S. K. . Emergence of californium as the second transitional element in the actinide series. Nat. Commun. 6, 1–8 (2015). 10.1038/ncomms7827 PMC4410632 25880116

[b15] Cross J. N. . Syntheses, structures, and spectroscopic properties of plutonium and americium phosphites and the redetermination of the ionic radii of Pu(III) and Am(III). Inorg. Chem. 51, 8419–8424 (2012). 22803932 10.1021/ic300958z

[b16] NSAC Isotopes Subcommittee, *Meeting Isotope Needs and Capturing Opportunities for the Future: The 2015 Long Range Plan for the DOE-NP Isotope Program*. (July 2015).

[b17] Miederer M., Scheinberg D. A. & McDevitt M. R. Realizing the potential of the actinium-225 radionuclide generator in targeted alpha particle therapy applications. Adv. Drug Deliv. Rev. 60, 1371–1382 (2008). 18514364 10.1016/j.addr.2008.04.009 PMC3630456

[b18] Kim Y.-S. & Brechbiel M. W. An overview of targeted alpha therapy. Tumor Biol. 33, 573–590 (2012). 10.1007/s13277-011-0286-y PMC7450491 22143940

[b19] Duckworth O. W., Bargar J. R. & Sposito G. Sorption of ferric iron from ferrioxamine B to synthetic and biogenic layer type manganese oxides. Geochim. Cosmochim. Acta 72, 3371–3380 (2008).

[b20] Lukens W. W., Shuh D. K., Schroeder N. C. & Ashley K. R. Identification of the non-pertechnetate species in Hanford waste tanks, Tc(I)-carbonyl complexes. Environ. Sci. Technol. 38, 229–233 (2004). 14740740 10.1021/es034318d

[b21] Jensen M. P., Neuefeind J., Beitz J. V., Skanthakumar S. & Soderholm L. Mechanisms of metal ion transfer into room-temperature ionic liquids: the role of anion exchange. J. Am. Chem. Soc. 125, 15466–15473 (2003). 14664592 10.1021/ja037577b

[b22] Spezia R., Beuchat C., Vuilleumier R., D'Angelo P. & Gagliardi L. Unravelling the hydration structure of ThX_4_ (X=Br, Cl) water solutions by molecular dynamics simulations and X-ray absorption spectroscopy. J. Phys. Chem. B. 116, 6465–6475 (2012). 22571631 10.1021/jp210350b

[b23] Atta-Fynn R. . Structure and hydrolysis of the U(IV), U(V), and U(VI) aqua ions from *ab initio* molecular simulations. Inorg. Chem. 51, 3016–3024 (2012). 22339109 10.1021/ic202338z

[b24] Bera M. K. . Aggregation of heteropolyanions in aqueous solutions exhibiting short-range attractions and long-range repulsions. J. Phys. Chem. C 120, 1317–1327 (2016).

[b25] Priest C., Tian Z. & Jiang D. First-principles molecular dynamics simulation of the Ca_2_UO_2_(CO_3_)_3_ complex in water. Dalton Trans. 45, 9812–9989 (2016). 26901265 10.1039/c5dt04576b

[b26] Zielinska B., Apostolidis C., Bruchertseifer F. & Morgenstern A. An improved method for the production of Ac-225/Bi-213 from Th-229 for targeted alpha therapy. Solvent Extr. Ion Exc. 25, 339–349 (2007).

[b27] Apostolidis C., Molinet R., Rasmussen G. & Morgenstern A. Production of Ac-225 from Th-229 for targeted α therapy. Anal. Chem. 77, 6288–6291 (2005). 16194090 10.1021/ac0580114

[b28] Teo B. K. EXAFS: Basic Principles and Data Analysis Springer (1986).

[b29] Stohr J. NEXAFS Spectroscopy **25** (Springer-Verlag Berlin Heidelberg, (1992).

[b30] Soderholm L., Williams C., Skanthakumar S., Antonio M. R. & Conradson S. The synthesis and characterization of the superconductor-related compound Pb_2_Sr_2_AmCu_3_O_8_. Z. Phys. B 101, 539–545 (1996).

[b31] Runde W. H. & Mincher B. J. Higher oxidation states of americium: preparation, characterization and use for separations. Chem. Rev. 111, 5723–5741 (2011). 21728323 10.1021/cr100181f

[b32] Ankudinov A. L., Ravel B., Rehr J. J. & Conradson S. D. Real space multiple-scattering calculation and interpretation of X-ray absorption near-edge structure. Phys. Rev. B 58, 7565–7576 (1998).

[b33] Lindqvist-Reis P. . The structures and optical spectra of hydrated transplutonium ions in the solid state and in solution. Angew. Chem. Int. Ed. Engl. 46, 919–922 (2007). 17200968 10.1002/anie.200603947

[b34] Stumpf T., Hennig C., Bauer A., Denecke M. A. & Fanghanel T. An EXAFS and TRFLS study of the sorption of trivalent actinides onto smectite and kaolinite. Radiochim. Acta 92, 133–138 (2004).

[b35] Allen P. G., Bucher J. J., Shuh D. K., Edelstein N. M. & Craig I. Coordination chemistry of trivalent lanthanide and actinide ions in dilute and concentrated chloride solutions. Inorg. Chem. 39, 595–601 (2000). 11229583 10.1021/ic9905953

[b36] Burns J. H. & Peterson J. R. The crystal structures of americium trichloride hexahydrate and berkelium trichloride hexahydrate. Inorg. Chem. 10, 147–151 (1971).

[b37] Downward L., Booth C. H., Lukens W. W. & Bridges F. A variation of the F-test for determining statistical relevance of particular parameters in EXAFS fits. AIP Conf. Proc. 882, 129–131 (2006).

[b38] Fried S., Hagemann F. & Zachariasen W. H. The preparation and identification of some pure actinium compounds. J. Am. Chem. Soc. 72, 771–775 (1950).

[b39] Kirby H. W. & Morss L. R. The Chemistry of the Actinide and Transactinide Elements Ch. 2 (Springer, Dordrecht (2006).

[b40] Shannon R. D. Revised effective ionic radii and systematic studies of interatomic distances in halides and chalcogenides. Acta Crystallogr. A32, 751–767 (1976).

[b41] Radchenko V. . Application of ion exchange and extraction chromatography to the separation of actinium from proton-irradiated thorium metal for analytical purposes. J. Chromatogr. A 1380, 55–63 (2015). 25596759 10.1016/j.chroma.2014.12.045

[b42] Horwitz E. P., McAlister D. R., Bond A. H. & Barrans R. E. Jr Novel extraction of chromatographic resins based on tetraalkyldiglycolamides: characterization and potential applications. Solvent Extr. Ion Exc. 23, 319–344 (2005).

[b43] Hagemann F. T. in *National Nuclear Energy Series, Manhattan Project Technical Section, Division 4: Plutonium Project*, Vol. 14A (eds Joseph J. K. and Seaborg G. T.), 14–44 (McGraw-Hill, 1954).

[b44] Karalova Z. K., Myasoedov B. F. & Rodionova L. M. Spectrophotometric determination of actinium. J. Anal. Chem. 28, 942–945 (1973).

[b45] Zachariasen W. H. Crystal chemical studies of the 5*f*-series of elements. I. New structure types. Acta Crystallogr. 1, 265–268 (1948).

[b46] Zachariasen W. H. Crystal chemical studies of the 5*f*-series of elements. VI. The Ce_2_S_3_-Ce_3_S_4_ type of structure. Acta Crystallogr. 2, 57–60 (1949).

[b47] Zachariasen W. H. Crystal chemical studies of the 5*f*-series of elements. XII. New compounds representing known structure types. Acta Crystallogr. 2, 388–390 (1949).

[b48] Farr J. D., Giorgi A. L., Bowman M. G. & Money R. K. The crystal structure of actinium metal and actinium hydride. J. Inorg. Nucl. Chem. 18, 42–47 (1961).

[b49] Weigel F. & Hauske H. The lattice constants of actinium(III) oxalate decahydrate. J. Less Common Metals 55, 243–247 (1977).

[b50] Stevenson P. C. & Nervik W. E. *The Radiochemistry of the Rare Earths, Scandium, Yttrium, and Actinium*. (National Academy of Sciences National Research Council (US). Subcommittee on Radiochemistry (1961).

[b51] Calvin S. XAFS for Everyone CRC Press (2013).

[b52] Ravel B. & Newville M. ATHENA, ARTEMIS, HEPHAESTUS: data analysis for X-ray absorption spectroscopy using IFEFFIT. J. Synchrotron Radiat. 12, 537–541 (2005). 15968136 10.1107/S0909049505012719

[b53] Kresse G. & Hafner J. *Ab initio* molecular dynamics for liquid metals. J. Phys. Rev. B 47, 558–561 (1993). 10.1103/physrevb.47.558 10004490

[b54] Perdew J. P., Burke K. & Ernzerhof M. Generalized gradient approximation made simple. Phys. Rev. Lett. 77, 3865–3868 (1996). 10062328 10.1103/PhysRevLett.77.3865

[b55] Kresse G. & Joubert J. From ultrasoft pseudopotentials to the projector augmented-wave method. Phys. Rev. B 59, 1758–1775 (1999).

[b56] Brendebach B. . X-ray absorption spectroscopic study of trivalent and tetravalent actinides in solution at varying pH values. Radiochim. Acta 97, 701–708 (2009).

[b57] Kirsh R. . Oxidation state and local structure of plutonium reacted with magnetite, mackinawite and chukanovite. Environ. Sci. Technol. 45, 7267–7274 (2011). 21755920 10.1021/es200645a

[b58] Marques Fernandes M., Scheinost A. C. & Baeyens B. Sorption of trivalent lanthanides and actinides onto montmorillonite: macroscopic, thermodynamic and structural evidence for ternary hydroxo and carbonato surface complexes on multiple sorption sites. Water Res. 99, 74–82 (2016). 27140904 10.1016/j.watres.2016.04.046

[b59] Galbis E. . Solving the hydratation structure of the heaviest actinide aqua ion known: the californium(III) case. Angew. Chem. Int. Ed. Engl. 122, 3899–3903 (2010). 10.1002/anie.200906129 20401881

[b60] Revel R. . First investigation on the L edges of the ^249^Cf aquo ion by X-ray absorption spectroscopy. Inorg. Chem. 38, 4139–4141 (1999).

